# Metabolites produced by probiotic Lactobacilli rapidly increase glucose uptake by Caco-2 cells

**DOI:** 10.1186/1471-2180-10-16

**Published:** 2010-01-20

**Authors:** Arun K Rooj, Yasuhiro Kimura, Randal K Buddington

**Affiliations:** 1 Integrative Biomedical Sciences, University of Alabama at Birmingham, Birmingham, AL 35205, USA; 2 Department of Food and Nutrition, Beppu University, 82 Kita-Ishigaki, Beppu, Oita 874-8501 Japan; 3 Department of Health and Sport Sciences, University of Memphis, Memphis, TN, 38152-3480, USA

## Abstract

**Background:**

Although probiotic bacteria and their metabolites alter enterocyte gene expression, rapid, non-genomic responses have not been examined. The present study measured accumulation of tracer (2 μM) glucose by Caco-2 cells after exposure for 10 min or less to a chemically defined medium (CDM) with different monosaccharides before and after anaerobic culture of probiotic *Lactobacilli*.

**Results:**

Growth of *L. acidophilus *was supported by CDM with 110 mM glucose, fructose, and mannose, but not with arabinose, ribose, and xylose or the sugar-free CDM. Glucose accumulation was reduced when Caco-2 cells were exposed for 10 min to sterile CDM with glucose (by 92%), mannose (by 90%), fructose (by 55%), and ribose (by 16%), but not with arabinose and xylose. Exposure of Caco-2 cells for 10 min to bacteria-free supernatants prepared after exponential (48 h) and stationary (72 h) growth phases of *L. acidophilus *cultured in CDM with 110 mM fructose increased glucose accumulation by 83% and 45%, respectively; exposure to a suspension of the bacteria had no effect. The increase in glucose accumulation was diminished by heat-denaturing the supernatant, indicating the response of Caco-2 cells is triggered by as yet unknown heat labile bacterial metabolites, not by a reduction in CDM components that decrease glucose uptake. Supernatants prepared after anaerobic culture of *L. gasseri, L. amylovorus, L. gallinarum*, and *L. johnsonii *in the CDM with fructose increased glucose accumulation by 83%, 32%, 27%, and 14%, respectively.

**Conclusion:**

The rapid, non-genomic upregulation of SGLT1 by bacterial metabolites is a heretofore unrecognized interaction between probiotics and the intestinal epithelium.

## Background

The interplay between the bacterial assemblages in the gastrointestinal tract (GIT) and the intestinal epithelium (microbial-epithelial "crosstalk") is an important determinant of host health and nutritional status. The interactions between pathogens and enterocytes activate signaling pathways that trigger disruption of the cytoskeleton and the tight junctions that link epithelial cells, alter expression of proinflammatory molecules, and stimulate secretion of fluid and electrolytes [1-4]. In contrast, members of the commensal gut flora that are considered as beneficial increase resistance to pathogens by modulating the host immune system and increase secretory IgA [5] upregulate expression of genes coding for mucin-2 (MUC-2) and human beta defensin-2 expression [6,7], compete with enteric pathogens for adhesion sites and nutrients [8], and produce bacteriocins [9,10]. Moreover the interactions between bacteria and enterocytes can elicit the synthesis of heat shock proteins [11], which up-regulate the activity of enterocyte glucose transporters [12] and modulate the activity of Na^+^/H^+ ^exchangers [13].

The influences of pathogens and beneficial bacteria on epithelial cells can be mediated by direct bacteria-cell contacts or indirectly via bacterial metabolites, such as toxins from pathogens [e.g., cholera toxin, *E. coli *heat stabile toxin) and short chain fatty acids from commensal bacteria (e.g., butyrate). Supplementing the diet with probiotic bacteria can increase small intestine absorption of nutrients [14-16] and electrolytes [17], and when added to culture media increase calcium uptake by Caco-2 cells [18]. Microarray analyses have revealed that long-term exposure to commensal bacteria and specific strains of probiotics (i.e., *Lactobacillus *GG) up-regulates genes involved in postnatal intestinal maturation, angiogenesis, and mucosal barrier functions, whereas genes associated with apoptosis and inflammation were down-regulated [19].

Absorption of glucose by enterocytes is mediated in part by the concentrative, high affinity, sodium-dependent glucose transporter (SGLT1), with rates of uptake dependent on the densities and activities of the SGLT1. Historically, studies of glucose uptake regulation have focused on the patterns of gene expression (genomic regulation), leading to changes in the abundances of transporter proteins. This include responses to bacterial lipopolysaccharides [20]. Enterocytes also have the ability to rapidly (<10 min) and reversibly regulate nutrient absorption independent of changes in the total cellular abundance of transporter proteins [21-24]. This non-genomic regulation of nutrient transporters allows enterocytes to adapt to the transient changes in luminal nutrient concentrations that occur before, during, and after the processing of meals.

Previous studies have reported the influences of probiotic bacteria on nutrient absorption, but have used prolonged periods of administration or exposure (6 h to days and weeks). As a result, the reported responses can be attributed to genomic regulation of the transporters. The present study demonstrates for the first time that metabolites produced by probiotic *Lactobacillus acidophilus *and four other species of *Lactobacilli *upregulate enterocyte glucose transport within 10 min of exposure using Caco-2 cells as a model for the intestine.

## Results

### Growth of Bacteria

**Figure 1:**
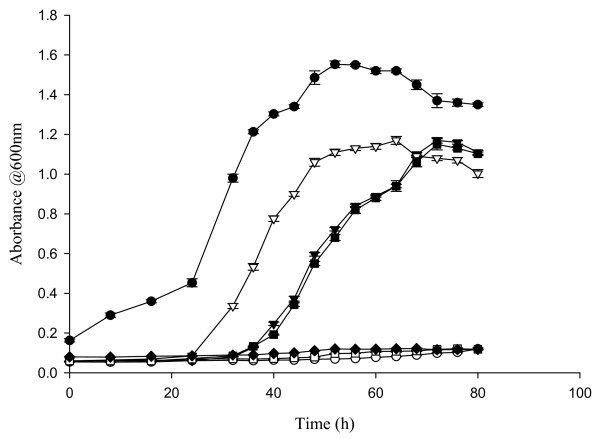
**Growth curves of *Lactobacillus acidophilus***. Growth curves of *Lactobacillus acidophilus *grown anaerobically at 37°C for 80 h in MRS broth (black circle) and in chemically defined media with glucose (inverted white triangle), fructose (inverted black triangle), mannose (black square), arabinose (white circle), ribose (white square), and xylose (black diamond) as the carbohydrate sources. Values are means ± SEM (n = 2 to 4).

Based on increases in absorption measured at 600 nm, the CDM-fructose and CDM-mannose elicited similar patterns of growth for *L. acidophilus *(Figure 1). However, after 80 h of anaerobic culture densities in CDM-fructose and CDM-mannose (10^8 ^CFU/ml) were lower compared to MRS broth (10^9 ^CFU/ml; P < 0.0001). Although CDM-glucose elicited an earlier increase in growth compared with CDM with fructose and mannose (shorter lag time), densities at 80 h were not higher compared with CDM-fructose and CDM-mannose cultures. The CDM alone or with arabinose, ribose, and xylose did not support the growth of *L. acidophilus*.

Growth curves for *L. acidophilus, L. amylovorus, L. gallinarum *and *L. johnsonii *cultured in the CDM-fructose were virtually identical (data not shown). Although the growth of *L gasseri *started earlier, the peak in absorption at 600 nm was achieved at about the same time as the other species.

### Glucose Uptake by Caco-2 Cells

**Figure 2:**
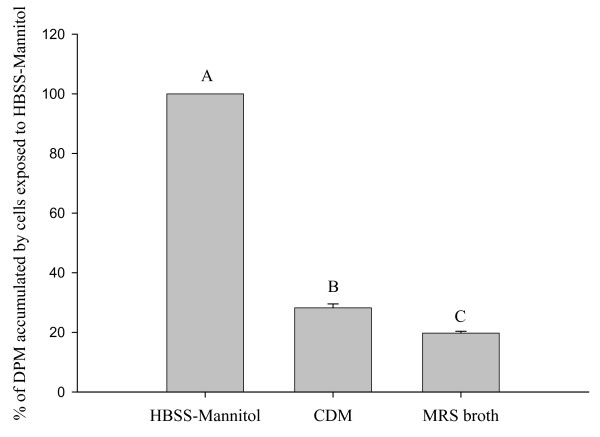
**Accumulation of tracer glucose by Caco-2 cells after exposure for 10 min to HBSS-mannitol (control), CDM without carbohydrate, and MRS broth**. Accumulation of tracer (2 μM) glucose by Caco-2 cells after exposure for 10 min to HBSS-mannitol (control), CDM without carbohydrate, and MRS broth. Values (means ± SEM) represent percentages of accumulation by cells on the same plate exposed to 25 mM HBSS-Mannitol (control). Bars with different letters are significantly different (n = 4 to 20 comparisons).

**Figure 3:**
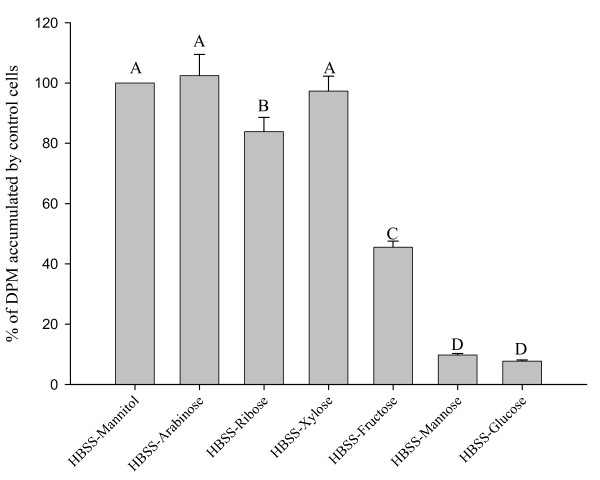
**Accumulation of tracer glucose by Caco-2 cells after exposure for 10 min to HBSS with 25 mM concentrations of different monosaccharides**. Accumulation of tracer (2 μM) glucose by Caco-2 cells after exposure for 10 min to HBSS with 25 mM concentrations of different monosaccharides. Values (means ± SEM) represent percentages of accumulation by cells on the same plate exposed to 25 mM HBSS-Mannitol (control). Bars with different letters are significantly different (n = 16 to 33 comparisons).

Exposure of the Caco-2 cells for 10 min to sterile MRS broth and to sterile CDM without carbohydrate decreased glucose accumulation by 91% and 82%, respectively, compared to cells exposed to the control solution (HBSS-Mannitol; P < 0.05) (Figure 2). Glucose accumulation by the cells also decreased (P < 0.05) when the 25 mM mannitol in the control HBSS was replaced by ribose (16% inhibition), fructose (55% inhibition), mannose (90% inhibition), and glucose (92% inhibition)(Figure 3). Replacement of mannitol by xylose and arabinose did not reduce glucose uptake. Based on these findings, CDM-fructose was selected as the carbohydrate source for the further studies because 1) it supported the growth of *L. acidophilus *and the other species of *Lactobacilli*, but 2) did not inhibit glucose accumulation by Caco-2 cells as much as the CDM with glucose or mannose.

### Exposure time and glucose uptake

**Figure 4:**
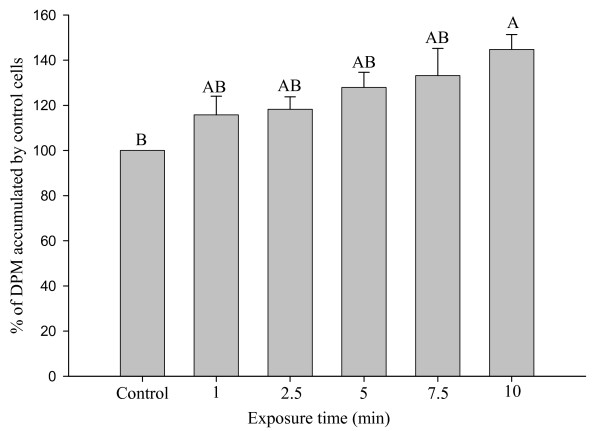
**Exposure time and glucose uptake**. Accumulation of tracer (2 μM) glucose by Caco-2 cells after exposure for 0 to 10 min to the cell-free supernatant of CDM-fructose after 72 h of anaerobic growth of *Lactobacillus acidophilus*. Values (means ± SEM) represent percentages of accumulation by control cells on the same plate that were exposed for 10 min to CDM-fructose (110 mM) that had not been used to culture bacteria. Bars with different letters are significantly different (n = 14 to 16 comparisons).

Glucose uptake by Caco-2 cells increased with longer exposures to the cell-free supernatant prepared after culturing *L acidophilus *for 72 h in CDM-fructose (110 mM) (Figure 4). Glucose uptake after a 10 min exposure to the supernatant was 40% higher compared with cells exposed to sterile CDM-fructose (110 mM) (P < 0.05).

Responses of Caco-2 cells to supernatants collected at different stages of bacterial growth

**Figure 5:**
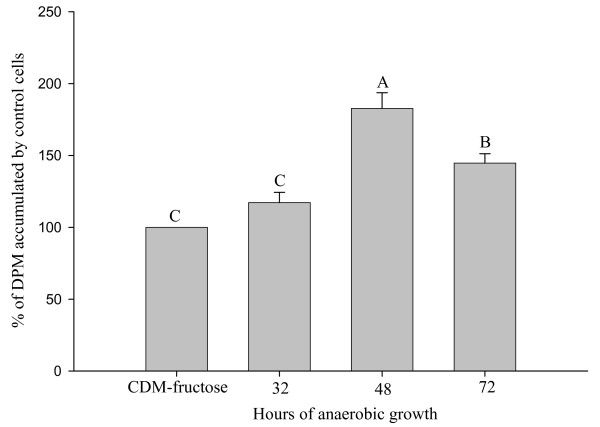
**Effect of supernatants collected at different stages of bacterial growth on glucose uptake**. Accumulation of tracer (2 μM) glucose by Caco-2 cells after exposure for 10 min to the cell-free supernatants prepared after 32 h (before exponential growth), 48 h (mid point of exponential growth), and 72 h (start of stationary phase) of anaerobic culture of *Lactobacillus acidophilus *in CDM with 110 mM fructose (CDM-fructose). Values (means ± SEM) represent percentages of accumulation by cells on the same plate exposed to CDM-fructose without bacteria. Bars with different letters are significantly different (n = 48 comparisons).

The supernatant prepared from CDM-fructose (110 mM) during the exponential phase of growth of *L. acidophilus *(48 h) resulted in the greatest increase in glucose uptake after a 10 min exposure compared with the sterile CDM-fructose (83%; P < 0.05; Figure 5). The supernatant collected at the stationary phase of growth (72 h) resulted in a 45% increase in uptake (P < 0.05), whereas the supernatant collected before exponential growth (32 h) did not elicit a significant increase in uptake.

### Responses of Caco-2 cells to heated supernatants

**Figure 6:**
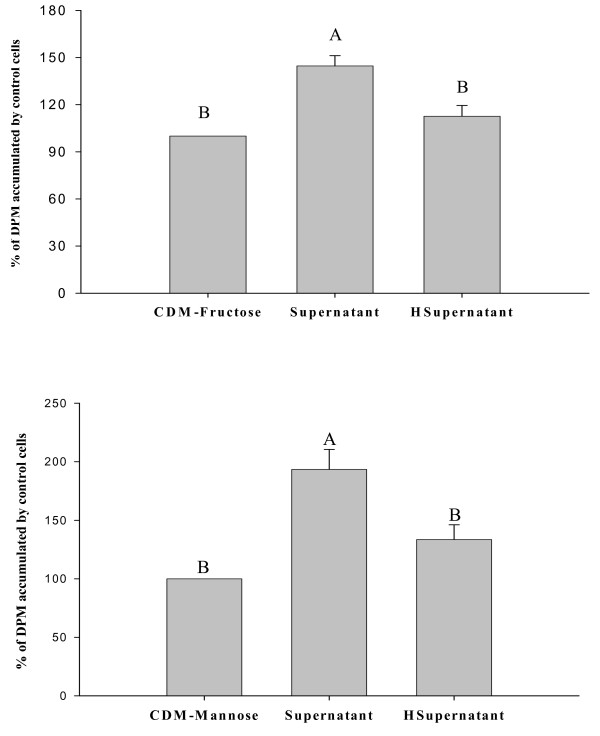
**Heated supernatants and glucose uptake**. Accumulation of tracer (2 μM) glucose by Caco-2 cells after exposure for 10 min to the unheated (Supernatant) and heated (100°C; 10 min; HSupernatant) cell-free supernatants prepared after 72 h of anaerobic growth of *Lactobacillus acidophilus *in CDM with 110 mM fructose (CDM-fructose; top panel) and 110 mM mannose (CDM-mannose; bottom panel). Values (means ± SEM) represent percentages of accumulation by cells on the same plate exposed to CDM-fructose without bacteria. Bars with different letters are significantly different (n = 8 to 12 comparisons).

Supernatants of CDM-fructose, and CDM-mannose harvested after 72 h of *L. acidophilus *growth increased glucose uptake by 40% and 93%, respectively, compared to Caco-2 cells exposed to the same media before the addition of bacteria (P < 0.05; Figure 6). In contrast, the corresponding heated supernatants caused a non-significant increase in glucose uptake by the cells.

### Response of Caco-2 cells to supernatants from the five species of *Lactobacilli*

**Figure 7:**
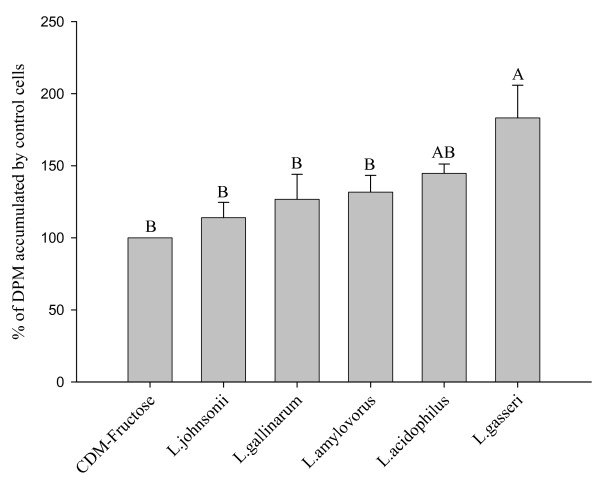
**Response of Caco-2 cells to supernatants from the five species of *Lactobacilli***. Accumulation of tracer (2 μM) glucose by Caco-2 cells after exposure for 10 min to the cell-free supernatants prepared after 72 h of anaerobic growth of five species of *Lactobacilli *in CDM-fructose (110 mM). Values (means ± SEM) represent percentages of accumulation by cells on the same plate exposed to CDM-fructose without bacteria. Bars with different letters are significantly different (n = 12 comparisons).

Rates of glucose uptake differed among Caco-2 cells exposed to supernatants prepared from CDM-fructose after 72 h of culturing the five species of *Lactobacilli*. All of the supernatants increased glucose uptake by the cells compared to the sterile CDM-fructose (P < 0.05; Figure 7). The greatest stimulation of glucose uptake was elicited by the supernatant prepared after growth of *L. gasseri *(83%), followed by *L. acidophilus *(45%), *L. amylovorus *(32%),*L. gallinarum *(27%), and *L. johnsonii *(14%).

## Discussion

The present findings demonstrate that metabolites produced by five species of *Lactobacilli *cultured anaerobically in a chemically defined medium cause a rapid increase in glucose uptake by Caco-2 cells. The response occurs too fast to be explained by the synthesis of new proteins and can therefore be considered as non-genomic. The increased uptake can be explained by the trafficking of existing transporters from a cytosolic source to the BBM or by the activation of transporters already present in the BBM. The rapid response to the metabolites resulting from the culture of probiotic bacteria is a novel finding and demonstrates a heretofore unrecognized interaction between probiotic bacteria and the intestine.

Glucose is transported across the BBM of enterocytes by a combination of SGLT1 and the low affinity, high capacity facilitative glucose transporter 2 (GLUT2) [25]. Since the uptake solution contained tracer concentration of glucose (2 μM) the majority of glucose accumulated by the Caco-2 cells would have been via SGLT1. There would be little or no uptake via the lower affinity GLUT2, which is dependent on a concentration gradient to drive absorption. This was verified in preliminary studies by the reduced accumulation of tracer glucose in the presence of phloridzin to inhibit SGLT1, but not when phloretin was used to inhibit GLUT2. Therefore, the increased accumulation of glucose by the Caco-2 cells was most likely dependent on higher densities or activities of SGLT1 in the BBM.

Exposure of the Caco0-2 cells for 10 min to the 110 mM glucose in MRS broth and the 25 mM in the HBSS-glucose depressed glucose uptake by 90%, whereas exposing the cells to mannose, ribose, and fructose to HBSS, which are not high affinity substrates for SGLT1, also inhibited glucose uptake by varying percentages. Similarly, SGLT1 mediated uptake of α-methyl-D-glucopyranoside by COS-7 cells is inhibited by exposure to fructose and mannose [26]. The lack of decline in glucose uptake after exposure of the cells to HBSS with arabinose, xylose, and mannitol corresponds with the negligible affinity of these sugars for SGLT1. Collectively, these findings indicate competition for SGLT1 transporter sites is partly responsible for the variable decreases in glucose accumulation by Caco-2 cells exposed to HBSS with the different monosaccharides or to the CDM with and without fructose. An alternative explanation is that the variation in glucose accumulation may be related to cellular metabolism of the monosaccharides. Specifically, enterocytes can transport and metabolize glucose, fructose [27], ribose [28], and mannose [29], all of which decreased glucose accumulation, despite the varying affinities for SGLT1. In contrast, absorption and metabolism of arabinose and xylose are limited, corresponding with a lack of influence on glucose accumulation. Although Caco-2 cells can metabolize glucose and fructose [30], which decrease glucose accumulation, we are unaware of information for the other sugars used in the present study. Enterocytes can metabolize other components of the CDM, notably amino acids. Hence, the 82% lower glucose uptake by the cells after exposure to carbohydrate-free CDM may be triggered by the metabolism of non-carbohydrate components of the CDM (e.g., amino acids) by the Caco-2 cells during the 10 min exposure.

The results from the heated supernatant address a critical concern that bacterial metabolism reduced or removed components of the CDM that reduce glucose accumulation or can be metabolized by Caco-2 cells (e.g., adenosine, glucose, amino acids). If this was so, glucose accumulation by Caco-2 cells would have been similar after exposure to the heated and unheated supernatants. Instead, glucose accumulation by Caco-2 cells was lower after exposure to the heated supernatant. This indicates that one or more heat labile bacterial metabolites are responsive for triggering a non-genomic increase in glucose uptake.

The bacterial metabolites responsible for the increased glucose uptake were not identified. Likely candidates include short chain fatty acids (SCFA), which are known to cause a genomic increase in the abundance and activity of SGLT1 and GLUT2 [31], the brush border membrane (BBM) Na^+^/H^+ ^exchanger 3 (NHE3) [32], and increase calcium absorption [18]. Polyamines are another category of bacterial metabolite that increase glucose transport by cultured enterocytes [33]. Because SCFA and polyamines are heat labile, concentrations in the heated supernatant would have been lower, corresponding with the reduced stimulation of glucose accumulation.

The types or proportions of metabolites produced vary during the different phases of bacterial growth. This is evident from greater increase in glucose uptake in response to supernatant collected during the exponential phase of *L. acidophilus *growth (83%) compared to the stationary phase (45%). Moreover, the present results suggest the types or proportions of metabolites produced vary among species of probiotic *Lactobacilli*. Specifically, the supernatant from *L. gasseri*, which grew faster and resulted in higher densities than the four other probiotic *Lactobacilli*, elicited the greatest increase in glucose accumulation; 83% increase relative to cells exposed to CDM before bacterial culture. Although the growth curves and peak densities for the four other species of *Lactobacilli *were virtually identical (data not shown), the resulting supernatants elicited varying increases in glucose accumulation (14 to 45% increase).

## Conclusions

The present findings indicate that unknown metabolites produced by probiotic *Lactobacilli *elicit rapid, non-genomic responses in the ability of intestinal epithelial cells to transport glucose. Whether genomic responses are also induced is unknown. The responses of Ca and Na uptake to bacterial metabolites (18,34) suggest the rapid stimulation of glucose transport triggered by the metabolites from *Lactobacilli *will be shared by carriers for other nutrients. There is an obvious need to identify the specific bacterial metabolites that elicit desired responses (i.e., increased nutrient absorption, immunomodulation, etc) and the bacterial species and conditions that promote the production.

## Methods

### Probiotic Bacteria Culture

A working culture of *L. acidophilus *(ATCC#4356) was propagated for 48 h at 37°C in DeMan, Rogosa and Sharpe (MRS) broth (Difco, Becton-Dickinson, Franklin Lakes, NJ) in a continuous shaker placed inside an anaerobic chamber with an atmosphere of 80% nitrogen, 10% carbon dioxide, and 10% hydrogen. The bacterial cells were sedimented by centrifugation (519 × g; 5 minutes) and were washed twice with sterilized water. The cells were suspended in a solution of 80% Dulbecco's Phosphate-Buffered Saline and 20% glycerol, and stored at -80°-C until used for experiments.

After characterizing a response of Caco-2 cells to the supernatant after culture of *L. acidophilus*, additional strains of *Lactobacilli *were obtained from Wyeth Nutrition (Collegeville, PA 19426, USA) for comparative purposes and working cultures were similarly prepared. These included *L. amylovorus *(ATCC#33620), *L. gallinarum *(ATCC#33199), *L. gasseri *(ATCC#33323), and *L. johnsonii *(ATCC#33200).

### Chemically Defined Media

The probiotic bacteria were cultured anaerobically to mimic conditions in the colon using a chemically defined medium (CDM; Table 1) [34] that was prepared without carbohydrate (pH = 6.5; 400 mOsm), filter sterilized (0.20 μm, Millipore, Billerica, MA), and stored at 4°C until used. A preliminary trial identified carbohydrates that would support the growth of *L. acidophilus *by adding arabinose, fructose, glucose, mannose, ribose, and xylose to the CDM at a concentration of 110 mM. Growth of *L. acidophilus *in MRS broth, which has 110 mM glucose, was used as a positive control. The CDM with different sources of carbohydrates and the MRS were pre-reduced and made anaerobic by placing them in the anaerobic chamber for 12-18 h before they were inoculated with the *L. acidophilus *suspension (200 μL with 10^9 ^CFU/ml in 500 ml). Aliquots were removed immediately after the inoculation and every 4 h thereafter during 80 h of anaerobic growth at 37°C and optical density at 600 nm was recorded to track bacterial growth and to define three different phases of the growth curves; the lag phase before rapid growth, at the middle of exponential growth, and after the start of the stationary phase. Additionally, after 80 h of culture (stationary phase), serial dilutions from each culture flask were plated on MRS agar that was pre-reduced by placing in the anaerobic chamber for 12-15 h before plating. After 3-4 days of anaerobic culture (37°C) the numbers of colony forming units (CFU/ml) on the plates were enumerated and were verified as *Lactobacillus spp. *based on colony morphology and Gram staining.

**Table 1:** Composition of the chemically defined medium (CDM) used to culture the Lactobacilli.

Component	(g/L)
Potassium hydrogen phosphate	3.1
di-ammonium hydrogen citrate	2.0
Potassium dihydrogen phosphate	1.5
Ascorbic acid	0.5
Potassium acetate	10
Tween 80 -	1.0
Heptahydrated magnesium sulphate	0.5
Hydrated manganese sulphate	0.02
Cobalt sulphate	0.5
Calcium Nitrate	1.0
Para-aminobenzoic acid	0.002
Biotin	0.01
Folic acid	0.002
Guanine	0.01
Thymine	0.1
Cytidine	0.1
2'-deoxyadenosine	0.1
2'-deoxyuridine	0.1

	(ml/L)

Non-Essential Amino Acids Solution^1^	500
Essential Amino Acids Solution^1^	63.5
Vitamin Solution^1^	200

^1 ^Purchased from Invitrogen, Carlsbad, CA

### Preparation of supernatants from the *Lactobacillus *spp. cultures

Based on the growth responses and reduced inhibition of glucose accumulation (see the Results section), *L. acidophilus *were cultured using CDM-fructose. Aliquots (100 ml) of the CDM-fructose medium were collected at the start of the growth phase (32 h), the mid point of the growth phase (48 h), and at the start of the stationary phase (72 h). For the remaining four species of probiotic *Lactobacilli*, aliquots of the culture medium were collected after 72 h of cultivation.

The culture media were centrifuged (11,180 × g; 15 min; 4°C) to sediment the bacteria. A portion of the cell-free supernatant was heated to 100°C in boiling water for 15 min to prepare a heated supernatant. The pH of the heated and unheated supernatants had declined to 4.3-4.5 and was adjusted to 7.4 with NaOH (10 M) to match the pH of the DMEM used to culture the Caco-2 cells. The osmolarity of the supernatants was measured (Wescor, Logan, UT) and was adjusted to 400 mOsm to similarly correspond with the DMEM. The heated and unheated supernatants were then filter sterilized (0.2 μm) and stored at 4°C until used (<1 week). The sedimented *L. acidophilus *after removal of the supernatant was suspended in HBSS with 25 mM mannitol to determine if direct interactions between the bacteria and the Caco-2 cells would alter glucose uptake.

### Glucose Uptake Assay by Caco-2 Cells

Caco-2 cells stably transfected to overexpress SGLT1 [35] (graciously provided by Dr. Jerrold R. Turner) were used between passages 22 to 30. Although Caco-2 cells are of colonic origin, they express enterocyte characteristics. Therefore, Caco-2 cells were considered a suitable model for obtaining insights into the non-genomic responses of the intestinal epithelium to bacterial metabolites. Following an established procedure, the cells were seeded in 24-well plates at a density of 10^5 ^cells/well and cultured at 37°C in a 5% CO_2 _and 95% air atmosphere using high glucose Dulbecco modified Eagle minimal essential medium (DMEM; Cellgro, Mediatech; Herndon, VA)] supplemented with heat inactivated calf serum (Gemini Bio-Products; West Sacramento, CA); 940 mM Na-bicarbonate; 20 mM HEPES, 200 mM, L-Glutamine, 100 mM Na-Pyruvate, and a combination of antibiotics [500 U/ml of both penicillin and streptomycin (Gemini Bio-Products), 10 mg/ml Tylosin (Sigma Chemical Co., St. Louis, MO), and 500 mg/ml Geneticin (USB Corporation, USA)].

Expression of SGLT1 by this line of Caco-2 cells does not require the cells to be confluent and can be induced by changing the culture medium from the high to low glucose DMEM supplemented with the same components. This was confirmed by a 90% decline in glucose accumulation when cells transferred to low glucose DMEM at 90% confluence were exposed to 0.5 mM phloridzin to inhibit SGLT1 mediated glucose uptake. The effect of carbohydrate source on glucose accumulation was evaluated by exposing Caco-2 cells at 90% of confluence for 10 min to CDM with and without the different sugars and to MRS broth. The control solution used to measure baseline glucose uptake consisted of HBSS (in mM: 137 NaCl, 5.4 KCl, 0.25 Na_2_HPO_4_, 0.44 KH_2_PO_4, _1.3 CaCl_2, _1.0 mM MgSO_4_, 4.2 NaHCO_3;_pH = 7.4) with 25 mM mannitol, which does not compete for the apical membrane glucose transporters and was used to balance osmolarity. All of the solutions were bacteria-free. After the 10 min exposure, the solutions were removed by aspiration and replaced with an uptake solution consisting of the control solution with tracer concentration (2 μM) of ^14^C-D-glucose (PerkinElmer Corp., Waltham, MA). The cells were allowed to accumulate the labeled glucose for 4 min. The uptake solution was removed, the cells were washed twice with 0.5 ml of cold (2-4°C) control solution, lysed with 0.1 N NaOH, and the cell lysates were collected, scintillant (Scintiverse, Fisher Scientific, USA) was added, and DPM of accumulated ^14^C D-glucose were measured by liquid scintillation counting.

The response of Caco-2 cells to the CDM after it had been used for bacterial culture was similarly evaluated. After overnight induction of SGLT1 expression, the cells were washed once with 37°C HBSS-Mannitol before adding 37°C control (HBSS with Mannitol) or treatment [unheated and heated supernatants after anaerobic culture of *Lactobacillus *in CDM-Fructose and CDM-Mannose (for comparative purposes)] solutions. After exposure to the solutions, glucose accumulation was measured as described above. Additional wells were exposed for 10 min to the resuspended *L. acidophilus *cells.

The influence of exposure period on glucose uptake was determined by exposing Caco-2 cells for 0, 1, 2.5, 5, 7.5 and 10 min to the cell-free supernatant prepared after culturing *L. acidophilus *in CDM-fructose for 72 h. A maximum exposure period of 10 min was used based on the maximal stimulation of glucose uptake by intact mouse intestine after a 10 min exposure to adenosine [23]. Moreover, 10 min was considered too short for a genomic response. Therefore, any changes in glucose accumulation would be caused by non-genomic mechanisms.

All comparisons were based on 4-6 wells per solution, and specific comparisons were performed on the same plate to avoid inter-plate and inter-day variation.

### Statistical Analysis

Rates of glucose accumulation (DPM/min) are presented as means ± SEM. One-way ANOVA was applied to search for an effect of treatment on glucose accumulation using the PROC GLM procedure of SAS (Version 9.1.3, SAS Institute Inc., Cary, NC,). When a significant treatment effect was detected, specific differences among treatments were identified by the Duncan's test. A critical value of P < 0.05 was used for all statistical comparisons.

## Authors' contributions

AR performed bacterial cultures, supernatant preparation, and measured glucose uptake by Caco-2 cells, YK participated in the design of the study and assisted with the glucose uptake studies, and RB helped in the conceptual design of the study, assisted with the analysis and interpretation of the data, helped with the preparation of the manuscript. All authors have read and approved the final manuscript.
